# Significance of intra-nodular vessel sign in differentiating benign and malignant pulmonary ground-glass nodules

**DOI:** 10.1186/s13244-021-01012-7

**Published:** 2021-05-26

**Authors:** Bin-jie Fu, Fa-jin Lv, Wang-jia Li, Rui-yu Lin, Yi-neng Zheng, Zhi-gang Chu

**Affiliations:** Department of Radiology, The First Affiliated Hospital of Chongqing Medical University, 1# Youyi Road, Yuanjiagang, Yuzhong District, Chongqing, 400016 China

**Keywords:** Ground-glass nodules, Vessel, Computed tomography, Differential diagnosis

## Abstract

**Background:**

The presence of pulmonary vessels inside ground-glass nodules (GGNs) of different nature is a very common occurrence. This study aimed to reveal the significance of pulmonary vessels displayed in GGNs in their diagnosis and differential diagnosis.

**Results:**

A total of 149 malignant and 130 benign GGNs confirmed by postoperative pathological examination were retrospectively enrolled in this study. There were significant differences in size, shape, nodule-lung interface, pleural traction, lobulation, and spiculation (each *p* < 0.05) between benign and malignant GGNs. Compared with benign GGNs, intra-nodular vessels were more common in malignant GGNs (67.79% vs. 54.62%, *p* = 0.024), while the vascular categories were similar (*p* = 0.663). After adjusting the nodule size and the distance between the nodule center and adjacent pleura [radius–distance ratio, RDR], the occurrences of internal vessels between them were similar. The number of intra-nodular vessels was positively correlated with nodular diameter and RDR. Vascular changes were more common in malignant than benign GGNs (52.48% vs. 18.31%, *p* < 0.0001), which mainly manifested as distortion and/or dilation of pulmonary veins (61.19%). The occurrence rate, number, and changes of internal vessels had no significant differences among all the pre-invasive and invasive lesions (each *p* > 0.05).

**Conclusions:**

The incidence of internal vessels in GGNs is mainly related to their size and the distance between nodule and pleura rather than the pathological nature. However, GGNs with dilated or distorted internal vessels, especially pulmonary veins, have a higher possibility of malignancy.

## Key points

- Pulmonary vessels are commonly detected in benign and malignant ground-glass nodules.
- The incidence of internal vessels in ground-glass nodules is mainly related to their size and distance between nodule and pleura.
- Ground-glass nodules with dilated or distorted internal vessels, especially pulmonary vein, have a higher possibility of malignancy.

## Introduction

An increasing number of pulmonary nodules, especially ground-glass nodules (GGNs), have been detected with the development of computed tomography (CT)-based screening for lung cancer in high-risk populations, which is extremely important for the prognosis of patients [1–4]. The qualitative diagnosis of pulmonary nodules depends on the display of their detailed manifestations. Thin-section CT (TSCT) reduces the partial-volume effect to some extent and provides more information by displaying the details of tissue anatomy and pathological changes [5, 6]. Hence, it can clearly show the relationship between the nodules and surrounding blood vessels and bronchi, improving the accuracy of diagnosis [7–9]. Therefore, TSCT plays a pivotal role in differentiating benign and malignant GGNs.

At present, there have been many studies on the diagnosis and differential diagnosis of GGNs, and the research on their morphological characteristics as obtained using CT were relatively sufficient [2, 9–14]. Several studies have suggested that exploring the relationship of pulmonary nodules and the surrounding vessels can help determine the nature of the lesions [8, 11, 15]. Gao F et al. believed that the presence of intact vessels passing through GGNs can be considered as a sign of independent blood supply in the lesions, and abnormal changes in blood vessels within the nodules indicate a greater likelihood of malignancy [8, 11].

Due to the diverse nature of GGNs and the common manifestation of internal blood vessels, there have only been a few studies on the significance of internal vessels in the diagnosis and differential diagnosis of GGNs. Further investigation concerning the relationship of GGN and pulmonary vessels, such as (1) the existence of differences in the presence of internal vessels and vascular changes between benign and malignant GGNs, (2) the existence of differences in vascular changes among GGNs with different pathological subtype, and (3) the factors that affect the presence of vessels in GGNs, would be beneficial. Therefore, the purpose of this study was to reveal the significance of pulmonary vessels in GGNs to facilitate the diagnosis and differential diagnosis of benign and malignant GGNs.

## Materials and methods

This retrospective study was approved by the ethics committee of our hospital, and the requirement for informed consent was waived.

### Patients

A retrospective data collection of patients with GGNs undergoing CT examinations in our hospital from January 2018 to January 2021 was performed. Cases that met the following conditions were included in this study: (1) nodules were manifested as GGN on the lung window; (2) GGNs were surgically resected and confirmed by pathological examination or disappeared during follow-up; and (3) time interval between CT examination and operation was less than 2 weeks. Patients with the following conditions were excluded from the study: (1) no thin-slice images with a thickness of ≤ 1 mm; (2) excessive solid components inside nodules affecting the observation of blood vessels; (3) image artifacts that affect the image analysis; and (4) incomplete clinical or imaging data. Finally, a total of 274 patients with 279 GGNs (malignant: 148 patients with 149 GGNs; benign: 126 patients with 130 GGNs) were enrolled in our study. Among malignant GGNs, 9 cases were pathologically diagnosed as atypical adenomatous hyperplasia (AAH), 31 cases were adenocarcinomas in situ (AIS), 70 cases were minimally invasive adenocarcinomas (MIA), and 39 cases were invasive adenocarcinomas (IA).

### CT examinations

The non-contrast chest CT scans were performed using Discovery CT 750 HD (GE Healthcare, Milwaukee, WI, USA), SOMATOM Perspective (Siemens Healthineers, Erlangen, Germany), and SOMATOM Definition Flash (Siemens Healthineers, Erlangen, Germany) CT scanner. All patients were placed in a supine position with raised upper limbs, and were asked to hold their breath after deep inspiration for better exposure. The scan range was from the entrance of the thorax to the costophrenic angle. The scanning parameters were as follows: tube voltage, 110–120 kVp; tube current, 80–250 mAs (using automatic tube current modulation technology); scanning slice thickness, 5 mm; reconstruction slice thickness and interval, 0.625 or 1 mm; matrix: 512 × 512; rotation time, 0.5 s; reconstruction algorithm, standard algorithm or medium-sharp algorithm.

### Image analysis

Two senior radiologists (Lv and Chu) evaluated the GGNs on axial, multi-planar reconstruction, and maximum intensity projection images without knowing the pathological results. All images were reviewed in the lung window setting (window level, − 600 HU; window width, 1500 HU). In case of disagreement, a consensus was reached after a joint discussion and/or consultation with a third senior radiologist.

**Fig. 1:**
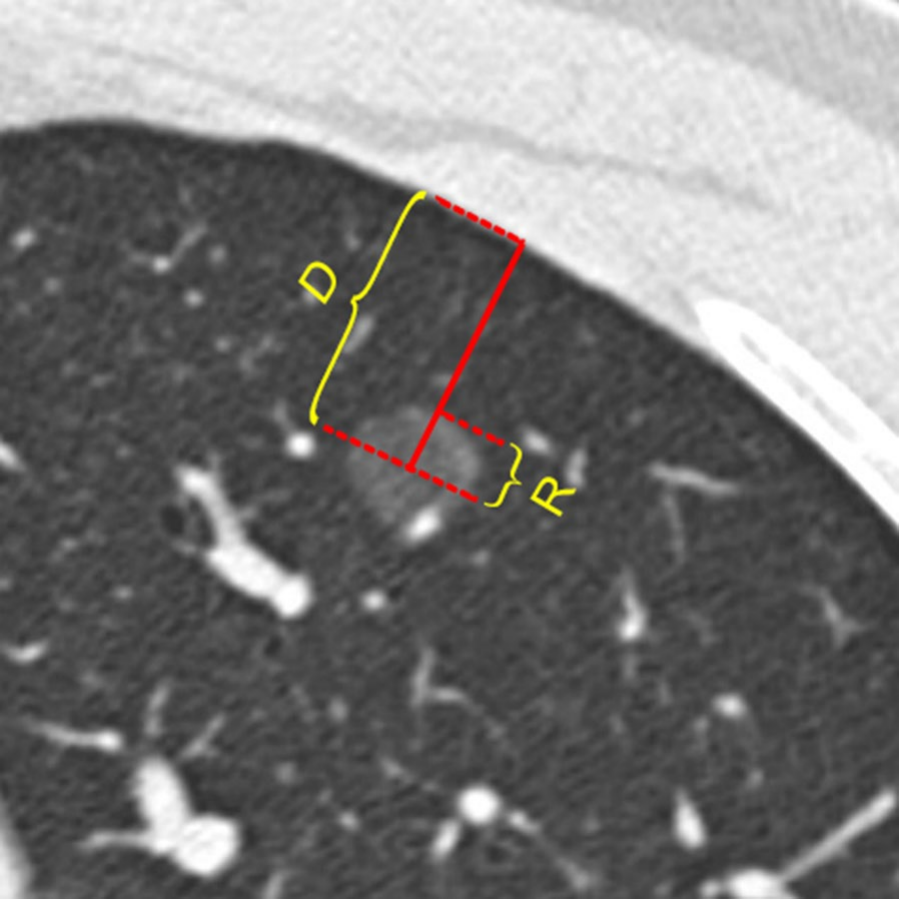
Definition of *RDR. D* was the shortest distance from the center of the nodule to the pleura;* R* was the radius of the nodule $\text{RDR} = \frac{R}{D}*100$

**Fig. 2:**
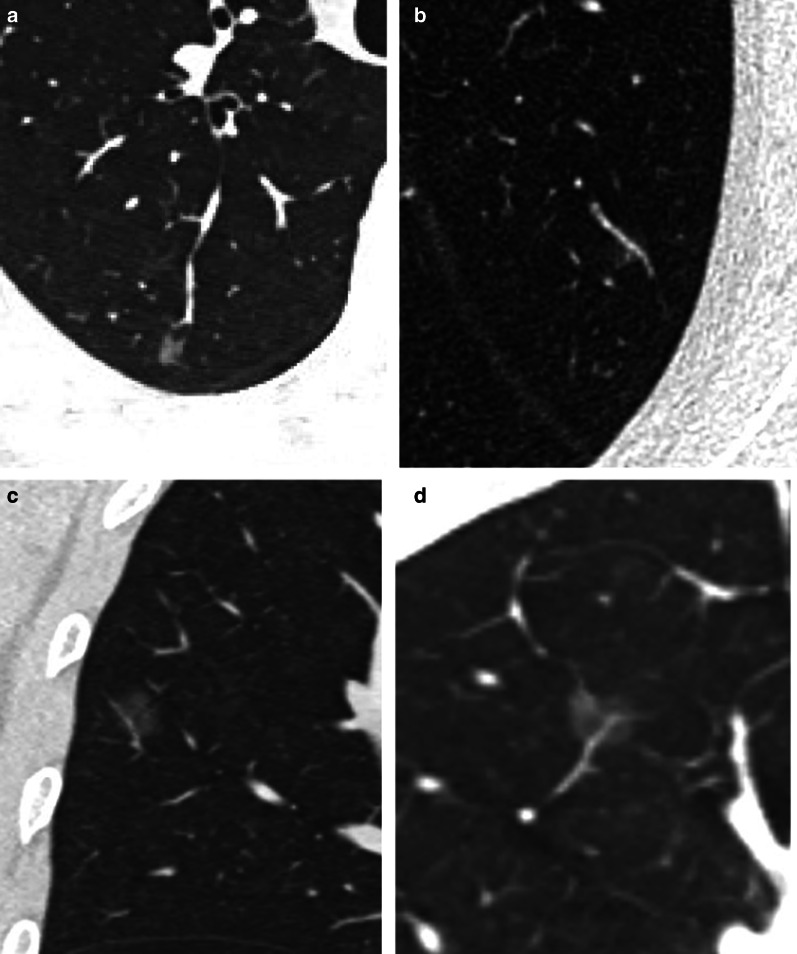
Computed tomography (CT) images of patients with benign ground-glass nodules (GGNs). A 5-mm GGN in the right upper lobe without internal vessels (**a**). A pulmonary vein (PV) branch without changes is detected in a 6-mm GGN in the left upper lobe (**b**) and a 9-mm GGN in the right upper lobe (**c**). A slightly dilated pulmonary artery (PA) branch is detected in a 9-mm GGN in the right upper lobe (**d**)

**Fig. 3:**
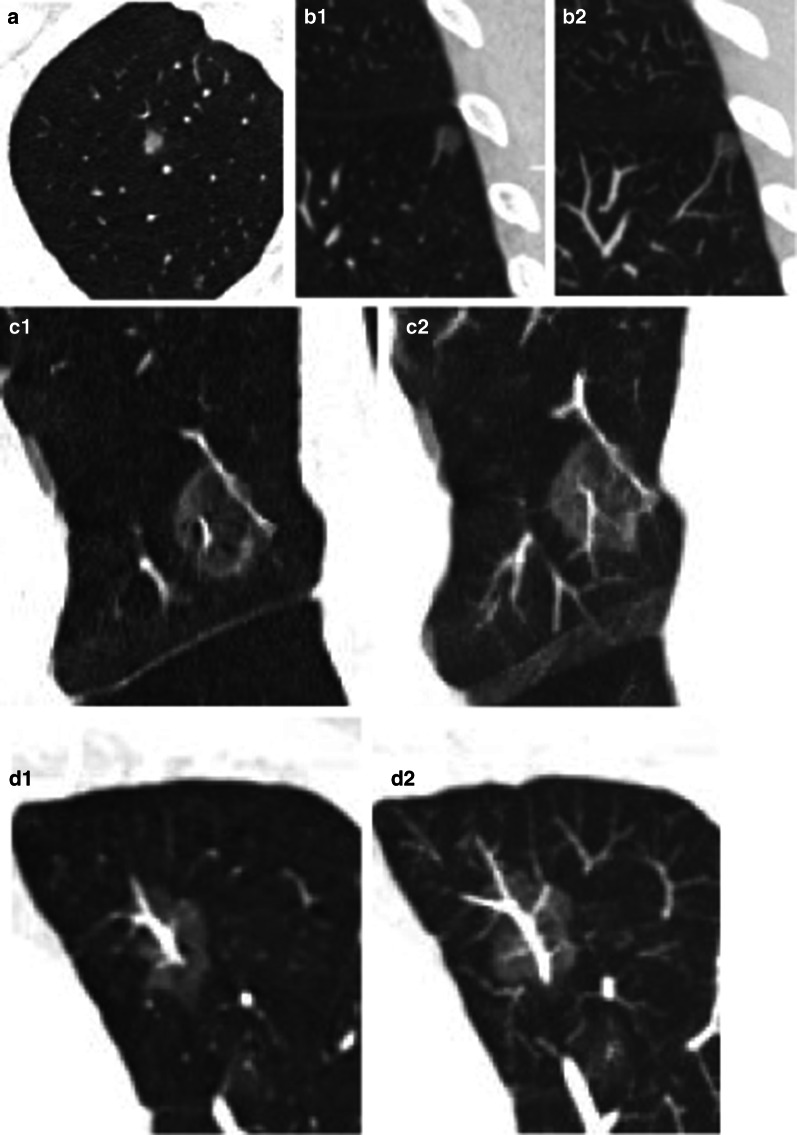
CT images of patients with malignant GGNs. A 6-mm GGN (atypical adenomatous hyperplasia) in the right upper lobe without internal vessels (**a**). A PA branch without change is detected in an 8.5-mm GGN [adenocarcinomas in situ (AIS)] in the left lower lobe (**b1**: **b2**). Two vessels (1 PA and 1 PV) were detected in a 17.5-mm GGN (AIS) in the left upper lobe (**c1**: **c2**), the PV was thickened with rigid course. An irregularly thickened PV branch is detected in a 19.5-mm GGN (IA) in the left upper lobe (**d1**: **d2**)

GGNs were evaluated in the following aspects: (1) the CT features of GGNs included: (a) size (the average of the longest diameter and the perpendicular diameter on axial images), (b) shape (round, oval, or irregular), (c) location (lung lobe and segment), (d) density (homogeneous or heterogeneous), (e) nodule-lung interface (well-defined or ill-defined), (f) margin (smooth, coarse), and (g) other manifestations (lobulation, spiculation, vacuole sign, pleural traction, air bronchogram, and containing solid components); (2) the positional relationship between the GGN and the pleura was expressed by a relative value of radius-distance ratio (RDR) (RDR = radius of nodule [*R*]/the shortest distance from the center of the nodule to the pleura [*D*] × 100%) (Fig. 1). When the RDR was closer to 100%, it indicated that the nodule was closer to the pleura; (3) the intra-nodular pulmonary vessels: (a) to determine whether there was blood vessel inside the nodules first (the wall of blood vessels was totally surrounded by GGNs, and the whole blood vessel in the GGN can be seen); (b) the number of blood vessels in the nodules; (c) the kind of blood vessels in the GGNs (pulmonary artery [PA] or the pulmonary vein [PV]); and (d) changes of intra-nodular vessels (no change (Figs. 2b, c, 3b); dilatation: the diameter of the intra-nodular segment was greater than that of the proximal segment before entering the lesion, or the intra-nodular vessel was significantly wider than other vessels at the same branch level; distortion: blood vessels deviated from the normal route [8]) (Figs. 2d, 3c, d).

### Statistical analysis

All statistical analyses were performed by using SPSS (version 21.0, IBM, NY, USA). Continuous data were expressed as mean ± SD, while categorical variables were expressed as numbers and percentages. The *t*-test was used to compare the age of patients with benign and malignant GGNs. The diameter and RDR of benign and malignant GGNs were compared by the Mann–Whitney *U* test. The different CT features of benign and malignant GGNs were compared by Pearson chi-square test or Fisher exact test. Pearson correlation analysis was used to analyze the correlation between the number of intra-nodular vessels and the diameter and RDR of nodules. The comparison of diameter and RDR among GGNs with different numbers of internal vessels was performed with the Kruskal–Wallis test. A *p*-value less than 0.05 indicates that the difference was statistically significant.

## Results

### Clinical characteristics of patients and CT features of GGNs

The clinical characteristics and CT features of GGNs of the patients are listed in Table 1. The diameter and RDR of malignant GGNs were greater (*p* = 0.003, *p* = 0.024), and the lobulated sign, the spiculated sign, pleural traction, well-defined border, and irregularity were more common in malignant nodules (*p* < 0.0001, *p* = 0.021, *p* = 0.008, *p* = 0. 025, and *p* = 0.002, respectively) compared with benign GGNs.

**Table 1:** Comparison of patients’ clinical characteristics and CT features of GGNs

Parameters	Malignant GGN (n = 149, 148 patients)	Benign GGN (n = 130, 126 patients)	*p* value
Age (year)	54.95 ± 11.82	53.73 ± 10.26	0.366
*Gender*			
Female	95 (64.19)	63 (50)	0.018
Male	53 (35.81)	63 (50)	
Diameter (mm)	10.84 ± 4.28	9.14 ± 3.68	0.003
RDR (%)	48.06 ± 29.05	40.35 ± 27.61	0.024
*Location*			
Right upper lobe	61 (40.94)	50 (38.46)	0.165
Right middle lobe	9 (6.04)	4 (3.08)	
Right lower lobe	18 (12.08)	29 (20.71)	
Left upper lobe	46 (30.87)	33 (25.38)	
Left lower lobe	15 (10.07)	14 (10.77)	
*Homogeneity*			
Homogeneous	22 (14.77)	30 (23.08)	0.075
Heterogeneous	127 (85.23)	100 (76.92)	
*Shape*			
Round/oval	37 (24.83)	55 (42.31)	0.002
Irregular	112 (75.17)	75 (57.69)	
*Nodule-lung interface*			
Well-defined	103 (69.13)	73 (56.15)	0.025
Ill-defined	46 (30.87)	57 (43.85)	
*Margin*			
Smooth	27 (26.21)	26 (35.62)	0.18
Coarse	76 (73.79)	47 (64.38)	
*Other signs*			
Lobulation	108 (72.48)	48 (36.92)	< 0.0001
Spiculation	15 (10.07)	4(3.08)	0.021
Vacuole	16 (10.74)	8 (6.15)	0.173
Pleural traction	13 (8.72)	2 (1.54)	0.008
Solid components	40 (26.85)	31 (23.85)	0.566
Air bronchogram	3 (2.01)	1 (0.77)	0.713

Data in parentheses are expressed as number (percentage) or mean ± standard deviation

CT, computed tomography; GGN, ground-glass nodule; RDR, radius-distance ratio

### The relationship between GGNs and pulmonary vessels

The conditions of vessels in GGNs are shown in Table 2. The internal vessels were more commonly detected in malignant GGNs than in benign ones (*p* = 0.024). There were no significant differences in the kind and number of intra-nodular vessels between benign and malignant GGNs (*p* = 0.346, *p* = 0.663). For GGNs with internal vessels, vessel changes were more common in malignant ones than in benign ones (*p* < 0.0001), especially the PV changes (*p* < 0.0001) (Fig. 3c, d).

**Table 2:** Conditions of vessels in GGNs

Parameters	Malignant GGN (n = 149)	Benign GGN (n = 130)	*p* value
*Intranodular vessel*			
Yes	101 (67.79)	71 (54.62)	0.024
No	48 (32.21)	59 (45.38)	
*Number of internal vessels*			
1	60 (59.41)	48 (67.61)	0.346
2	30 (29.70)	19 (26.76)	
3	9 (8.91)	4 (5.63)	
≥ 4	2 (1.98)	0 (0)	
*Kinds of internal vessels*			
PA	34 (33.66)	27 (38.03)	0.663
PV	29 (28.71)	22 (30.99)	
PA + PV	38 (37.62)	22 (30.99)	
*Vascular changes*			
Without significant changes	48 (47.52)	58 (81.69)	< 0.0001
Distorted/dilated	53 (52.48)	13 (18.31)	
PA distorted/dilated	14/72 (19.44)	10/49 (20.41)	0.896
PV distorted/dilated	41/67 (61.19)	4/44 (9.09)	< 0.0001

Data in parentheses are expressed as number (percentage)

GGN, ground-glass nodule; PA, pulmonary artery; PV, pulmonary vein

The diameters and RDRs corresponded with the benign and malignant GGNs considering that their size and RDR may have differences (Table 3). There were no significant differences in the presence of internal vessels between benign and malignant GGNs (each *p* > 0.05) within different diameter and RDR ranges.

**Table 3:** Presence of internal vessels in different diameter and RDR ranges

Range	Malignant GGN	Benign GGN	*p* value
*Diameter (mm)*			
< 10	38/78 (48.72)	33/86 (38.37)	0.182
10–15	33/40 (82.50)	26/31 (83.87)	1.000
16–20	24/25 (96.00)	9/10 (90.00)	0.496
> 20	6/6 (100.00)	3/3 (100.00)	-
*RDR (%)*			
0–24	17/37 (45.95)	13/45 (28.89)	0.111
25–49	35/50 (70.00)	32/49 (65.31)	0.618
50–74	20/24 (83.33)	11/14 (78.57)	1.000
75–100	29/38 (76.32)	15/22 (68.18)	0.492

Data in parentheses are expressed as number (percentage)

RDR, radius-distance ratio; GGN, ground-glass nodule

**Fig. 4:**
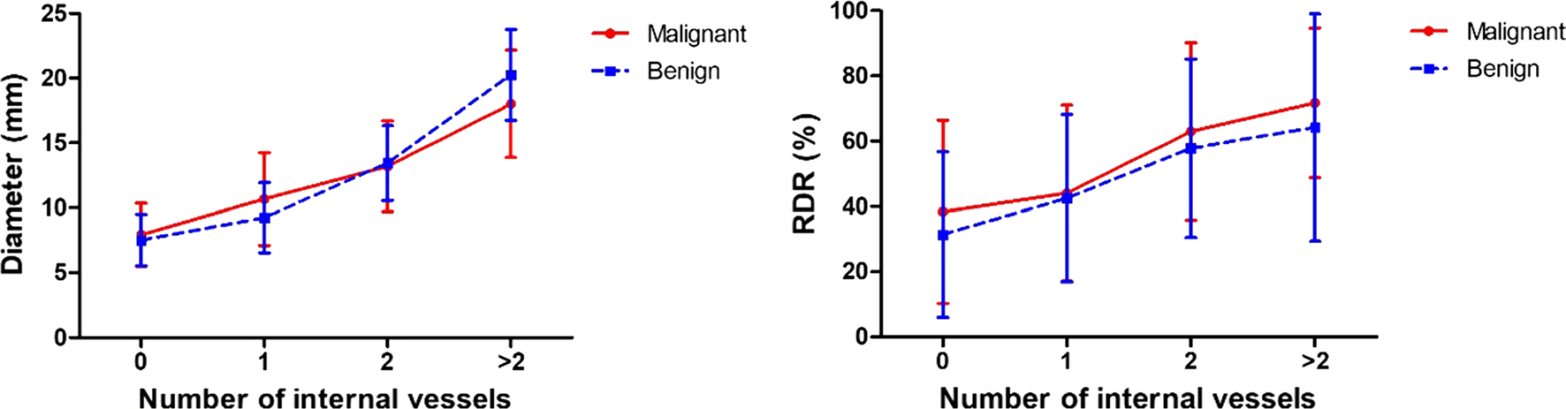
Correlations between diameter and RDR of GGNs and numbers of intra-nodular vessels

The correlations between diameter and RDR of GGNs and numbers of intra-nodular vessels are shown in Fig. 4.

The number of internal vessels increased along with the diameter and RDR, and the differences were significant among each group (each *p* < 0.0001). In addition, the number of internal vessels was positively correlated with diameter and RDR The correlation coefficients were 0.655 (*p* < 0.0001) and 0.379 (*p* < 0.0001) in malignant GGN, and for benign ones, they were 0.722 (*p* < 0.0001) and 0.283 (*p* = 0.017) respectively.

### Internal vessels in different pathological subtypes of GGNs

The relationship between vessel and different pathological types of malignant GGNs are shown in Table 4. There were no significant differences in the occurrence of internal vessels, the number of intra-nodular vessels, nodules with vessel changes, and PV changes among the different pathological subgroups or between the pre-invasive group (AAH and AIS) and the invasive group (MIA and IA) (each *p* > 0.05).

**Table 4:** The relationship between vessel and different pathological types of malignant GGNs

	AAH (n = 9)	AIS (n = 31)	MIA (n = 70)	IA (n = 39)	P1	P2
With internal vessel(s)	5 (55.56)	20 (64.52)	45 (64.29)	31 (79.49)	0.287	0.403
*Number of internal vessels*						
1	4 (80.00)	15 (75.00)	27 (60.00)	14 (45.16)	0.521	0.112
2	1 (20.00)	4 (20.00)	13 (28.89)	12 (38.71)		
≥ 3	0 (0)	1 (5.00)	5 (11.11)	5 (16.13)		
Vascular changes	2 (40.00)	10 (50.00)	21 (46.67)	20 (64.52)	0.423	0.605
Changes in PV	2 (40.00)	8 (40.00)	14 (31.11)	18 (58.06)	0.137	0.853

Data in parentheses are expressed as number (percentage). P1 was the comparison between each pathological subgroup. P2 was the comparison between the pre-invasive group (AAH and AIS) and the invasive group (MIA and IA)

AAH, atypical adenomatous hyperplasia; AIS, adenocarcinoma in situ; MIA, minimally invasive adenocarcinoma; IA, invasive adenocarcinoma; PV, pulmonary vein

## Discussion

Pulmonary GGN is an early manifestation of various benign and malignant lesions; thus, early diagnosis determines the way of treatment, which influences the patients’ prognosis [1, 16–19]. Several studies have revealed that benign and malignant GGNs can be distinguished according to their specific CT morphological features, such as the size, lobulation, spiculation, vacuoles, pleural traction, and air bronchogram sign [17, 20–23]. According to the present results, it was found that benign and malignant GGNs had significant differences in size, shape, boundary, pleural traction, lobulated sign, and spiculation in terms of morphology, which corroborated the previous results. However, none of the mentioned CT features above is specific for differentiating them. Therefore, it was necessary to find other valuable clues for diagnosing and differentiating GGNs.

The present study confirmed that pulmonary vessels were commonly detected in GGNs, especially in malignant ones, while the kinds of intra-nodular vessels were similar between benign and malignant GGNs, consistent with previous results [8, 11]. However, the difference in the proportion of internal vessels between benign and malignant GGNs did not exist after matching the diameter and distance between the nodule and pleura (RDR). Thus, the presence of internal vessels is a common phenomenon in GGNs, and the occurrence and numbers of intra-nodular vessels were only related to their size and location. It is apparent that the larger the diameter of the nodules, the greater its coverage, and the possibility of covering pulmonary vessels is also greater. In addition, the closer the nodule is to the pleura, the greater the likelihood of covering vessels as there is relatively more branches of pulmonary vessels near the pleura. Therefore, it cannot be simply considered that the higher rate of internal vessels in malignant GGNs was related to the increased demand for blood supply [8], or the intra-nodular vessel was a specific indicator for distinguishing GGNs.

For GGNs with internal vessels, vessels with abnormal changes were more commonly detected in malignant ones, which was consistent with previous studies [8, 10, 11]. Small blood vessels may be distorted, rigid, or aggregated when the tumor tissue infiltrates and grows into the bronchus-vascular bundle or the interlobular septa, or when the proliferated fibrous components pull the surrounding structures [24–26]. In addition, the growth and metabolism of tumor tissue are higher than that of normal tissues; hence, it requires more blood supply, which may cause the dilation of blood vessels [8]. Previous studies have shown that when a PV was involved by SPN, it was highly suspected to be malignant due to the possibility of PV participating in the blood supply of the tumor [27–29]. However, this study revealed that the kind of internal vessels was of little significance in distinguishing GGNs. In contrast, intra-nodular PV with abnormal changes was mainly detected in malignant GGNs, which may be attributed to the relatively thinner wall besides its richer oxygen content. Thus, the PV change is more meaningful for differentiating the benign and malignant GGNs. In addition, the Fleischner Society guidelines mention that routine follow-up of a single GGN smaller than 6 mm is not recommended [30]. However, in this study, it was found that internal vessels were also commonly detected in GGNs less than 6 mm in diameter, and one of them with vascular changes was confirmed as MIA. Therefore, the present result indicated that the conditions of internal vessels of GGNs smaller than 6 mm should be evaluated before making a strategy for further treatment, if there are internal vascular changes, especially venous changes, were detected in such nodules, routine follow-up is still needed for them.

Among malignant GGNs with different pathological nature, the relationships between pulmonary vessels and nodules were similar, which was inconsistent with previous results that the number of intra-nodular vessels was related to the malignant degree of GGNs [12]. In their study, the volume of different GGNs was significantly different, which may have affected the results to some extent. According to the previous research focusing on sub-centimeter GGNs or pure GGNs [11, 31], the pathological types of malignant GGNs were closely related to the nodule-vascular relationship. Thus, the relationship between GGNs and pulmonary vessels is worthy of further study.

This study has two limitations. First, some mixed GGNs with more solid components were not included in this study due to the inability to properly evaluate the internal blood vessels, which may lead to selection bias. Second, there were no suitable quantitative indicators for the morphological changes of intra-nodular vessels.

## Conclusion

In conclusion, intra-nodular vessels are commonly detected in GGNs; the incidence of internal vessels in GGNs is mainly related to their size and distance between nodule and pleura rather than their pathological nature. GGNs with dilated or distorted internal vessels, especially PV, have a higher possibility of malignancy. However, the relationship between pulmonary vessels and nodules is not significantly related to the aggressiveness of the nodules.

## Data Availability

The datasets generated and/or analyzed during the current study are not publicly available because the cases are from the Picture Archiving and Communicating System of our Hospital but are available from the corresponding author on reasonable request.

## Abbreviations

AAH: Atypical adenomatous hyperplasia
AIS: Adenocarcinoma in situ
GGN: Ground-glass nodule
IA: Invasive adenocarcinoma
MIA: Minimally invasive adenocarcinoma
PA: Pulmonary artery
PV: Pulmonary vein
RDR: Radius–distance ratio
TSCT: Thin-section computed tomography
